# Advancements in Nanodrug Delivery Systems as Controlled-Release Systems for Glaucoma Therapy: An Inspirational Step Toward Translation from Research to Clinic

**DOI:** 10.3390/biomedicines14051137

**Published:** 2026-05-18

**Authors:** Tanin Hosseinkhani, Ahmad Karami, Shahla Mirzaeei, Ali Nokhodchi

**Affiliations:** 1 Nano Drug Delivery Research Center, Health Technology Institute, Kermanshah University of Medical Sciences, Kermanshah 6715847141, Iran; taninhosseinkhani@gmail.com; 2 Pharmaceutical Sciences Research Center, Health Institute, Kermanshah University of Medical Sciences, Kermanshah 6715847141, Iran; ahmadkarami202@gmail.com; 3 School of Life Sciences, University of Sussex, Brighton BN1 9QJ, UK

**Keywords:** ocular drug delivery systems, nanocarriers, glaucoma, nanodrug delivery, sustained release, translation from research to clinic

## Abstract

Glaucoma is a collection of disorders that result in permanent vision loss and is characterized by a gradual decline in retinal ganglion cells. While it may not always be high, intraocular pressure (IOP) is the sole risk factor that can be modified according to extensive clinical research. Glaucoma remains the leading cause of irreversible blindness, yet early treatment lowering intraocular pressure is effective in slowing the rate of visual deterioration. Issues like poor absorption, low bioavailability, and short drug resistance time have thus made the management of glaucoma challenging when using conventional ophthalmic drugs. Thus, extensive research has been conducted to explore specific nanodrug delivery systems from various nanocarriers such as nanoparticles, micelles, liposomes and nanofibers, with a focus on systems that have achieved drug release for more than 12 h. These carriers have demonstrated substantial improvements in a lot of the evaluated aspects: enhancing ocular barrier-crossing capabilities, improving bioavailability, prolonging drug release, targeting active tissues of interest, and reducing IOP. This review covers recent developments in nanocarrier ocular delivery systems regarding the management of glaucoma. In this study, the advantages and disadvantages of each system were evaluated and their potential for advancing translation from research to clinic were assessed.

## 1. Introduction

Glaucoma is a complex and chronic eye disease that poses a significant challenge in the field of ophthalmology. Prolonged elevated IOP can cause irreversible optic nerve damage and result in vision loss. Approximately 3.5% of individuals aged 40 to 80 globally are believed to have glaucoma [1]. By 2040, it is expected that 111.8 million individuals will be affected by glaucoma [2]. Glaucoma, being a chronic condition, requires prolonged administration of anti-glaucoma medications, hence making patient adherence a crucial factor in achieving optimal treatment outcomes. The predominant ocular drug delivery methods currently include topical eye drops and traditional formulations like ointments. Conventional delivery systems encounter several drawbacks, including rapid drainage through nasolacrimal routes, leading to limited bioavailability. This necessitates frequent administration, resulting in increased exposure to side effects. In the past decade, novel drug delivery systems with sustained- and extended-release properties have gained significant attention. Particularly systems with extended-release formulations exceeding 12 h, which help reach the required and adequate drug concentration by a single administration in a day.

Prolonged elevated IOP can cause irreversible optic nerve damage and result in vision loss. The local management of glaucoma comprises eye drops, but they are limited due to their drawbacks, such as the frequent application due to the short retention time on the ocular surface. This comprehensive review investigates advanced ocular drug delivery nanocarriers designed to sustain drug release for durations exceeding 12 h, with a particular focus on glaucoma treatment. In addition to providing a detailed classification of novel drug delivery systems, the study also evaluated the outcomes of these technologies through preclinical animal models, critically assessing the physicochemical characteristics and tissue compatibility of the selected formulations.

### 1.1. Glaucoma

Statistics indicate that approximately 70 million individuals globally are affected by glaucoma [3], which is the primary cause of permanent vision loss [4]. Glaucoma is a group of progressive ocular conditions, primarily caused by elevated intraocular pressure (IOP). This heightened pressure can gradually damage the optic nerve, ultimately resulting in irreversible blindness. Glaucoma has many types, based on the degree of its presence and cause. The two major forms of glaucoma are primary open-angle and closed-angle glaucoma, which have the characteristic feature of optic nerve damage due to an increased IOP. The first one is more prevalent, slowly progressive, and leads to peripheral vision loss initially, progressing gradually to tunnel vision. On the other hand, closed-angle glaucoma is abrupt in onset, with eye pain, blurred vision, redness of the eye, and other vision changes. Others are normal pressure glaucoma, whereby IOP is within normal limits but there is damage to the optic nerve probably due to reduced blood supply to the optic nerve; pigmentary glaucoma, which is a form of open-angle glaucoma caused by the dispersion of the iris’ pigmented cells throughout the eye; and uveitic glaucoma results from changes in aqueous humor production and outflow, leading to alterations in the anterior chamber structure of the eye [5].

Glaucoma can further be classified according to the cause of the problem, where it is divided into primary and secondary. The cause of primary glaucoma is not established yet. However, secondary glaucoma tends to have causes such as advanced cataracts, inflammation, and diabetes. As with many other medical conditions, the development of glaucoma is linked to several risk factors, such as racial background, the long-term use of corticosteroids, hyperthyroidism, and larger corneal thickness [6].

### 1.2. Pathology of Glaucoma

**Figure 1:**
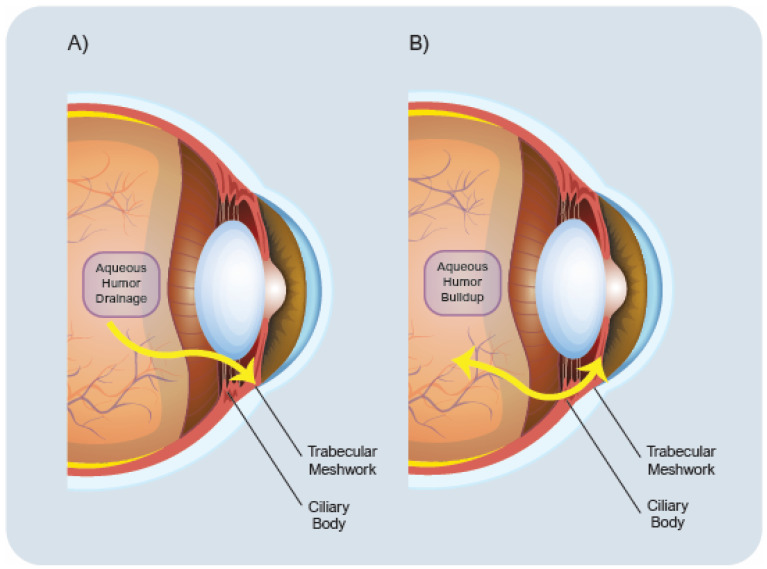
Aqueous humor drainage mechanism: (**A**) normal eye; (**B**) glaucoma.

Glaucoma pathophysiology involves the control and drainage of the aqueous humor, secreted by the ciliary bodies lying in the anterior chamber of the eye, between the lens and the iris. Normally, a significant volume of the aqueous humor drains, passing through the trabecular meshwork and Schlemm’s canal and entering the episcleral veins. Furthermore, the sclera absorbs a small percentage of aqueous humor. Aqueous humor permeates through the fibers of the ciliary body, originating from the intercellular spaces to maintain intraocular pressure within normal limits (Figure 1). The aqueous flow is controlled by the trabecular meshwork, regulating the outflow resistance through intercellular communication and extracellular matrix modulation. Failure in this process leads to an increase in resistance to the outflow of aqueous humor, which consequently results in an increased IOP [7]. Metabolic alterations play a critical role in the pathology of glaucoma, encompassing a spectrum of dysfunctions such as mitochondrial dysfunction oxidative stress, impaired glucose metabolism, and dysregulation of lipid metabolism [8]. These disruptions are key drivers in the development and progression of the disease, ultimately leading to energy deficiencies and damage to the neurons within the optic nerve. Given the important role those metabolic pathways play in the pathophysiology of glaucoma, they represent promising targets for therapeutic interventions aimed at limiting the detrimental effects of these alterations. Glaucoma is defined by increased IOP and ongoing damage to retinal ganglion cells. As a result, existing treatment methods mainly aim to lower intraocular pressure to help slow the advancement of the disease. This focus has contributed to the extensive use of standard pharmacological therapies, especially topical forms like eye drops.

### 1.3. Glaucoma Treatment

The medication for glaucoma is customized for each patient according to the form of glaucoma, its severity, the root cause, and risk factors, if any [9]. The treatment options utilized in the management of glaucoma consist of laser therapy, surgical interventions, and pharmacotherapy. Medicinal management of glaucoma has the core approach of reducing intraocular pressure via a wide variety of classes of drugs; these involve beta-blockers, adrenergic agonists, carbonic anhydrase inhibitors, prostaglandin analogs, and cholinergic agonists (Table 1). These drugs relieve intraocular pressure either by increasing the outflow of aqueous humor or by decreasing its production. Combination therapy with these drugs is also performed in most cases of glaucoma management, and many combined formulations are available in the market for better results [5]. Drugs with varying pharmacologic mechanisms typically exhibit at least partially additive effects in reducing IOP in glaucoma treatment. Timolol and other β-adrenergic antagonists exhibit synergistic IOP-lowering effects when used alongside miotic medications, prostaglandin analogs, α2-agonists, and carbonic anhydrase inhibitors. Enhanced patient compliance due to a decrease in the number of medications used, negating the necessity to administer two distinct medications 5 to 10 min apart to avoid a washout effect from the subsequent drug, improved safety by reducing exposure to the BAK preservative, and financial savings for the patient by eliminating a need to pay for two different medications, are among the benefits of combination treatments [10].

## 2. Ocular Drug Delivery Limitations

Most medications are administered to the eye using topical formulations, with eye drops accounting for approximately 90% of ocular products available in the market, including those for glaucoma treatment [11]. Despite their extended use, medicine administration via eye drops encounters several problems owing to the unique anatomy and physiology of the eye. The limited precorneal surface limits the volume (30 microliters) of eye drops placed on the ocular surface. Furthermore, the loss of a large amount of eye drops due to blinking and tear turnover [11]. The special structure of tears that has three different layers, including hydrophilic and lipophilic compounds, Lipids, proteins and enzymes, which can create a barrier against the penetration of the drug or even cause the degradation of the drug [12]. It has been shown that 60% of the active drug is washed out within two minutes post-application; a similar pattern is observed with almost all the active substances being removed from the surface of the cornea within 15–25 min [13]. This may, therefore, require an increased frequency of drug administration throughout the day to affect the desired therapeutic effect. This greatly affects patient compliance since the increased frequency of application is less convenient and thus less acceptable to the patients [5]. Furthermore, the combination of various conditions (such as heart disease, glaucoma, and dementia) and sociocultural factors (like living alone) may lead to a decline in patients’ compliance.

Moreover, the use of preservatives in conventional formulations is yet another limitation in ocular drug delivery. Preservatives maintain drug sterility and microbiological integrity; while various preservatives are available, benzalkonium chloride (BAK), a quaternary ammonium compound, is the most widely used in ophthalmic formulations [14]. In both experimental and clinical studies, most preservatives have shown significant toxic effects on the ocular surface. These effects are attributed to three main mechanisms: disrupting the tear film’s lipid layer, directly harming corneal and conjunctival epithelial cells, and inducing immune-allergic reactions [15,16]. Additionally, if preservatives enter the anterior chamber, they can cause endothelial cell loss [17]. Several studies have identified preservatives as contributors to ocular surface inflammation, allergies, fibrosis, dry eye syndrome, and other conditions. Frequent dosing weakens the epithelium’s ability to maintain the tear film, potentially leading to ocular surface disease. This risk is heightened for patients who require multiple eye medications, have chronic eye conditions like dry eye or glaucoma, or need postoperative medication [15].

## 3. Ocular Drug Delivery Systems

### 3.1. Conventional Ocular Drug Delivery Systems

Soluble eye drops and suspensions, emulsions, and ocular ointments are the most common ocular drug delivery systems in use. Ophthalmic suspension drops are formulated especially for lipophilic drugs, such as erythromycin, and have the advantages of a longer shelf life than solutions. Nonetheless, there is a critical limitation regarding the size of the drug particles; larger particles can cause discomfort and induce reflex tearing. Hence, drug particles must be uniformly small, complicating the production process [18]. Ophthalmic emulsions, like suspensions, enhance drug shelf life and are appropriate for lipophilic drugs. Their formulation, however, which incorporates surfactants, is associated with eye irritation and congestion, casting doubt on their safety [19]. Ointments and gels have been developed as alternatives to the deficiencies of eye drops. Its viscosity and slower ocular clearance rate enhance the drug’s retention and absorption in the eye. Though having these advantages, these forms can lead to blurred vision, flaky eyelashes, secretion of increased tears, and other signs of preservative toxicity, such as foreign body sensation, stinging–burning, and dry eye, and their use is largely restricted to night applications [13,20]. Even though they are commonly utilized, traditional methods for delivering drugs to the eyes come with notable drawbacks, such as limited bioavailability, quick drug elimination, and low patients’ compliance. These issues highlight the necessity for innovative delivery approaches that can overcome ocular obstacles and offer prolonged therapeutic benefits.

### 3.2. Novel Ocular Drug Delivery Systems

Recent advances in nanotechnology and biomaterial sciences have allowed the development of improved drug delivery systems designed to overcome the limitations of conventional formulations [11]. These new systems are tissue-compatible, minimally immunogenic, non-toxic, and result in easy penetration of various components across the cornea to reduce the frequency of required dosing, thus increasing patient compliance. The primary objective of this novel system is to improve the bioavailability and increase the life of drugs by overcoming the major drawbacks of ocular drug delivery methods [5]. Some of the new drug delivery technologies under study for use in the eye include the following: inserts, nanofibers, liposomes, dendrimers, nanoparticles, and in situ gelling systems. Some of the studies that have analyzed modern ocular drug delivery systems are works by Zhai [21], Kompella [22], and Patel [23]. These studies reveal the potential and the effectiveness of these new delivery systems in improving therapeutic outcomes for eye diseases. These works set the course of ocular pharmacotherapy for the future.

A focused review on nanoformulations used solely for ocular delivery in glaucoma treatment was conducted by Zhai et al. in 2021 [21]. The reviewed work comprises research on the use of niosomes, liposomes, nanoparticles, and dendrimers as novel approaches to enhance the efficiency of drug delivery to the eye. Notably, the review did not cover other forms of advanced drug delivery systems, such as nanofibers, and inserts, and also did not assess the drug release, resistance time and pharmacokinetics in the eyes [21].

In 2020, a comprehensive review of ocular drug delivery systems was conducted by Kompella et al., categorizing them based on the anatomical location of drug delivery into ocular surface, periocular, and intraocular drug delivery systems. The focus of this research primarily centered on the effects and formulations of the bimatoprost drug, with limited examination of the drug release duration from the designed formulations [22].

Following a similar investigative path, Patel et al., in a 2022 review article, expanded upon the categorization of modern ocular drug delivery systems. They further divided the systems into four distinct groups: inorganic-based carriers, contact lens-based delivery systems, hydrogel-based, and polymer-based systems. Unlike the Kompella study, pharmacokinetics and the duration of the drug release from these systems were not a primary focus of Patel’s work, again illustrating the consistent gap in the literature regarding the length of time a drug remains effective following administration in ocular therapies [23]. These studies do not systematically evaluate drug delivery systems based on their ability to provide sustained therapeutic release for clinically relevant periods. In contrast, our review looks at ocular delivery systems that can maintain drug release for over 12 h and also their potential for translation to the clinic. This approach allows for a comparison of different delivery systems and offers a more practical view on improving treatment adherence and therapeutic effectiveness in chronic ocular diseases.

**Figure 2:**
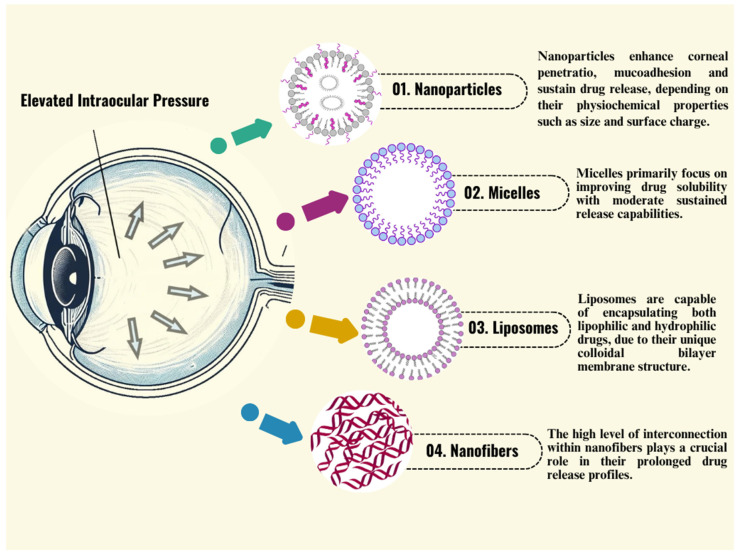
Novel anti-glaucoma nanocarriers.

In this research, the novel ocular drug delivery nanocarriers were explored, particularly focusing on nanoparticles, micelles, liposomes and nanofibers capable of controlling drug release for durations exceeding 12 h, highlighting the animal studies with a specific emphasis on glaucoma treatment (Figure 2). The research examined the results of these technologies using preclinical animal models, carefully evaluating the physicochemical properties and tissue compatibility of the chosen formulations. A targeted literature search was conducted using the PubMed and ScienceDirect databases to identify relevant studies published between January 2013 and March 2026. The search was performed in January of 2025 and April of 2026 using combinations of keywords including “ophthalmic nanoformulation,” “novel ocular drug delivery systems,” “controlled-release ophthalmic drug delivery system,” “ocular nanoparticles,” “ocular liposomes,” and “ocular micelles.” These terms were combined using Boolean operators (AND, OR) to refine the search results. Inclusion criteria comprised original research articles and review papers focusing on ocular drug delivery systems with an emphasis on nano-based or sustained-release formulations. Studies not related to ocular applications, articles lacking sufficient experimental details and drug release less than 12 h were excluded. The initial search results were screened based on titles and abstracts to remove irrelevant studies, followed by full-text evaluation of the remaining articles. Additional relevant studies were identified through the reference lists of selected papers. This study, apart from classifying the various available modern delivery systems, also evaluated the outcomes of the technologies through animal studies, the physicochemical properties and tissue compatibility studies of the selected formulations were critically assessed. The benefits and drawbacks of each innovative system have also been analyzed. The importance of innovative drug delivery strategies in glaucoma emphasizes the urgent need for these emerging ocular delivery systems to progress toward clinical evaluation and large-scale industrial translation.

#### 3.2.1. Nanoparticles

Nanoparticles used for drug delivery can range in size from 50 to 500 nm to effectively overcome the challenges of ocular drug delivery. The absorption and resistance of nanoparticles in the eye are significantly influenced by their surface charge. Positively charged particles are known to adhere to the surface of the eye longer and penetrate more deeply than negatively charged particles [24]. This is due to the negatively charged nature of the corneal and conjunctival tissues. Ophthalmic nanoparticles are typically designed as either nanospheres or nanocapsules. Nanocapsules are vesicle-like structures where the drug is enclosed within a central cavity [25], whereas nanospheres are part of matrix systems in which the drug and polymer are evenly distributed throughout the particle [24]. Current methods for preparing nanoparticles involve the use of toxic organic solvents, and the scaling up of their production to an industrial level is also a problem. Additionally, nanoparticles suffer from low stability and the risk of drug leakage from their structures [23].

For the preparation of ophthalmic nanoparticles that enable extended drug release, a variety of polymers have been utilized, including polymethacrylates (Eudragit) [26], polyethylene glycol (PEG), polycaprolactone (PCL) [27,28], gelatin [29,30], ceria [31], polyvinylpyrrolidone (PVP) [32], chitosan, carbopol [33,34], alginate [34,35,36], and Poly (lactic-co-glycolic acid) (PLGA) [37]. These studies are documented and described in Table 2.

Among the studies conducted on the subject of ocular nanoparticles with extended drug release for the treatment of glaucoma, nanoparticles containing brimonidine [27], timolol [29], pilocarpine [31], melatonin [37], betaxolol [38], dorzolamide [39], and brinzolamide [40] were highlighted, noting their ability to sustain release for over 12 h.

In 2020, Sharma et al. [27] developed brimonidine-encapsulated nanoparticles using a blend of Vitamin E-TPGS (Tocopheryl polyethylene glycol succinate) and PCL to improve penetration of the drug for ophthalmic use. The average diameter of the nanoparticle was approximately 243.4 nm in size, with a zeta potential of around +3.23 mV. Following their preparation, the nanoparticles were integrated into an in situ gelling system formulated with Poloxamer 407, facilitating the in vitro release of 93.91% of the encapsulated drug within the first 24 h. Further transcorneal permeation experiments on goat eyes demonstrated that the drug’s release rate from the nanoparticle-containing gelling system was significantly higher than that from conventional eye drops, achieving 51.18 µg cm^−2^ h^−1^ compared to 7.618 µg cm^−2^ h^−1^ for eye drops. Rabbit eye’s study results indicated that the system decreased eye pressure by approximately two-fold compared to eye drops over a 6 h period. This enhanced performance is attributed to the gelling system’s superior adhesion to the ocular surface and the nanoparticles’ improved penetration through the cornea. Sensitivity tests conducted on rabbits’ eyes showed the system did not cause corneal or iris damage for three days, indicating potential safety and effectiveness for use in the enhancement of ocular drug delivery [27]. Hydrogel-based systems for ocular drug delivery that incorporate nanoparticles offer an effective approach for prolonged drug release by merging the extended residency time of hydrogels with the drug-loading and permeation benefits of nanoscale carriers. In these formulations, the hydrogel matrix functions as a depot that prevents quick clearance by tears, while the nanoparticles embedded within act as additional reservoirs, creating a dual-barrier release mechanism that supports sustained drug delivery. Mechanical characteristics like viscoelasticity and crosslink density are important as they affect ocular retention, patient comfort, and the kinetics of drug release. These hybrid systems have shown enhanced bioavailability in comparison to traditional formulations by improving corneal contact time and preserving therapeutic drug levels for longer durations. Recent improvements in hydrogel-based ocular drug delivery systems highlight the importance of stimuli-responsive mechanisms. These include pH, temperature, and ion-triggered gel formation, along with adjustable mechanical properties that affect drug release and retention. These technologies work well with nanocarrier-based strategies, especially in situ gelling systems and nanofiber-based platforms, where controlled drug release and material properties are crucial for providing long-term therapeutic benefits. Hydrogel nanoparticles made from polyvinylpyrrolidone (PVP) improve the ability of drugs to permeate the blood–retinal barrier, making them particularly suitable for managing posterior segment diseases such as age-related macular degeneration (AMD) [41].

In 2017, researchers utilized PCL to fabricate nanospheres and nanocapsules for the ocular delivery of pilocarpine and found that the drug loading capacity of the nanocapsules was nearly three times higher than that of the nanospheres. In vitro release tests revealed that pilocarpine was released from the nanocapsules over a period of 40 days, whereas 85% of the drug contained within the nanospheres was released within 6 days. Notably, a greater initial burst release of the drug was observed from the nanospheres compared to nanocapsules. The prolonged release from nanocapsules can be attributed to their thicker shell, which effectively entraps the drug within the core, acting as a barrier to drug release. These mechanisms of drug release include the following: the immediate diffusion that contributes to the initial burst release, and then the barrier and erosion, both contributing to the sustained release. The cytotoxicity test, using bovine corneal endothelial cells, assessed the biocompatibility of these nanoparticles, and no significant cell death or morphological changes were observed up to a concentration of 5000 µg/mL. A slight reduction in metabolic activity in cells has been observed at higher concentrations than 1000 µg/mL. The nanoparticle biocompatibility studies were performed in vivo by injecting the nanoparticles with a buffer into the anterior eye chamber. No turbidity, irritation, or edema was noted at the end of 42 days of the experiment. The corneal thickness revealed no significant difference compared with the blank control, indicating it to be of high biocompatibility. The therapeutic efficacy of these nanoformulations was then explored in sixteen New Zealand white rabbits with experimentally induced glaucoma. In the glaucoma animal models, α-chymotrypsin was administered by injection into the posterior chamber of the eye. Following the administration of α-chymotrypsin for 1 to 2 weeks, two drops of tobramycin–dexamethasone ophthalmic solution and one drop of diclofenac sodium ophthalmic solution were instilled three times daily to prevent ocular inflammation and discomfort. A surgical procedure was conducted on one eye of the rabbits. In the two experimental groups of animals (six rabbits per group), PILO-PCL NSs or PILO-PCL NCs were administered into the anterior chamber of the eyes. IOP was maintained within the therapeutic range by nanocapsules for 42 days, while nanospheres achieved such an effect only for 6 days. Such data are in good accordance with the in vitro release studies and confirm the potential for using nanocapsules in ocular drug delivery systems for a long-lasting effect [28].

In 2018, Shokry et al. [29] prepared gelatin-based nanoparticles for ocular delivery of timolol. A double desolvation method was used with glutaraldehyde as the cross-linking agent to increase particle stability. Gelatin type A was dissolved in pure deionized water at 40 °C with continuous gentle stirring until completely dissolved. Subsequently, acetone was introduced to the solution to facilitate the initial desolvation and rapid sedimentation of high molecular weight gelatin. The supernatant, which contained low molecular weight gelatin, was discarded, and the residual sediment was re-dissolved in purified deionized water, with or without 0.5% Timolol Maleate. The gelatin solution was subsequently desolvated with acetone. To solidify the nanoparticles, various ratios of glutaraldehyde were included to crosslink the particles. The obtained nanoparticles showed an average diameter and zeta potential of 205 nm and 12.5 mV, respectively. In vitro release studies revealed the existence of an initial burst release of drug molecules, possibly located at the nanoparticle surface, followed by a more gradual and sustained release. In this way, the drug was released for up to 25 h, resulting in sustained therapeutic effects. In vivo studies carried out in rabbit eyes aimed at studying the pressure-lowering effect confirmed that the system could reduce IOP up to 25 h, and during the first 10 h, the pressure reduction was more significant. Further, such a prolonged period of reduced pressure would prevent the occurrence of neuronal damage associated with elevated levels of the IOP. The Draize test for the safety of the nanoparticles showed no sensitivity reaction for up to 21 days. However, slight irritation was observed after 1 h of application because of the preservatives in the formulation. Further histological examinations also support the safety of the nanoparticles, confirming a tissue state that would confirm the potential of these gelatin-based nanoparticles for effective and safe ocular delivery [29].

In 2020, Pérez et al. [30] developed gelatin-based nanoparticles encapsulating the drug timolol through the ethanol–water solvent displacement method. The resulting nanoparticles were then suspended in a viscous solution of HPMC (Hydroxypropyl methylcellulose) to enhance application in the eye. A particle size of 193 nm and a slightly negative value of the zeta potential of −0.684 mV indicated stability and a possible application in ocular tissues. The in vitro drug release pattern showed that 80.46% of timolol was released within the first 24 h, and then there was a more gradual release of the remaining drug in the next three days, thus securing a sustained delivery system. Biocompatibility studies were carried out by means of MTT assays on human corneal epithelial cells and turned out to be more biocompatible for the first 24 h compared to conventional timolol eye drops; cell viability decreased further with increasing concentrations of timolol. In vivo studies regarding IOP reduction using rabbits resulted in a reduction of 30.09% for an 8 h profile for the nanoparticles. In contrast, timolol eye drops achieved a 21.11% reduction in the same duration. Incorporation of nanoparticles into an HPMC solution increased the duration of pressure reduction to 12 h and extended the IOP reduction. A tolerance study using blank nanoparticles (without timolol) at a gelatin concentration of 30 mg/mL indicated good ocular tolerance in rabbits, with no observed discomfort or inflammation of the eyelid after 3, 6, and 24 h [30].

In 2019, Luo et al. [31] explored the potential of ceria nanoparticles for delivering pilocarpine with a focus on enhancing drug delivery through the ocular barrier. By functionalizing these nanoparticles with chitosan and ZM241385, these materials were able to open corneal epithelial tight junctions, thereby improving drug transport into the eye tissue. Additionally, such nanoparticles with special design also showed anti-inflammatory and antioxidant properties in both in vitro and in vivo studies, offering potential benefits in reducing the damage of glaucoma. The addition of chitosan and ZM241385 had marked impacts on the physical features of the nanoparticles. The developed nanoparticles had a zeta potential between 12.5 and 36.3, due to the anionic nature of ZM241385, indicating stability and therefore making them quite potential for interaction with negatively charged cell surfaces. The study aimed to point out the ability of the nanoparticles to control drug release and sustain release over 7 days. For in vivo evaluation, the ceria nanoparticles reduced the raised IOP and retained the IOP within the therapeutic range for 3 days in rabbit models with ocular hypertension; this is a significant improvement compared to conventional drops, whose effect was retained only for 4 h. This research thus evidences the potential of functionalized ceria nanoparticles with chitosan and ZM241385 for improved ocular delivery, sustained therapeutic effects, and extra protective benefits against glaucoma-related damage [31].

In 2016, Agban et al. [32] developed ophthalmic nanoparticles for pilocarpine release using a combination of Zinc oxide (ZnO) with PVP, then cross-linked with collagen for the developed nanoparticles to improve mechanical properties like degree of swelling and tissue adhesion. In vitro release studies revealed that drug release was sustained for a period of 14 days with initial burst release due to drug adsorbed on the nanoparticle surface, followed by a prolonged release phase. Having a size of less than 20 nm, facilitated NPs potential for efficient ocular delivery. The zeta potential of these ZnO/PVP nanoparticles measured 23.2 mV, which would be suitable for cross-linking in collagen, since the latter is negatively charged in nature. On top of that, the release rate of ZnO from these nanoparticles remains below the IC50 value, proposing their safety in ophthalmic applications [32].

In a 2013 study, Kunal et al. [38] developed a chitosan nanoparticle-based carrier for delivering betaxolol hydrochloride in ocular applications, which was intended to enhance the corneal permeability and raise the pre-corneal residence time. The formation of the nanoparticles was based on the spontaneous emulsification method. Particle sizes were in the range of 168–260 nm, while the zeta potential was found in the range of 25.2–26.4 mV. The in vitro testing in simulated tear fluid showed a biphasic drug release that comprised an initial fast release followed by a more sustained release over 12 h. The ocular tolerance of the nanoparticles was assessed using the Hen Egg-Chorion Allantoic Membrane technique, which proved to be non-irritant. Stability tests of the nanoparticles indicated that no notable change in particle size or drug content after 3 months of storage at 25 ± 2 °C and 60 ± 5% relative humidity was observed. In vivo pharmacodynamic studies conducted on rabbits with dexamethasone-induced glaucoma showed that the nanoparticles effectively reduced IOP more significantly than current marketed formulations at the end of 5 h [38].

In 2020, Zhou et al. [42] developed lipid–polymer nanoparticles (LPNs) as carriers of brinzolamide (Brz) for sustained drug delivery to improve corneal permeation and local therapeutic efficacy. Brz-LPNs are composed of PLGA nanocores loaded with Brz (Brz-NPs) enveloped by a lipid shell. This resulted in an optimal mean particle size at 151.23 ± 1.64 nm, a high EE percentage of 86.7 ± 2.28%. The zeta potential measured for the Brz LPNs was 7.53 ± 0.52 mV. Successful entrapment of Brz within the LPNs was further confirmed with FTIR. The amount of Brz released from the LPNs was also very well sustained compared to AZOPT^®^ and Brz lipid particles (LPs), while after 12 h, only 83.2% of the drug was released, whereas nearly 100% release was shown by the free drug. In addition to that, the relative cumulative corneal permeability of Brz from LPNs was much higher compared to the commercially available AZOPT^®^. In vivo, it was shown that Brz-LPNs at a dose of 1 mg/mL of Brz, lower the IOP more effectively and for a longer time as compared to AZOPT^®^ at 10 mg/mL of Brz over a period of 24 h [42].

Silk fibroin is a natural protein polymer of light and heavy chains and is synthesized mainly by silkworms of the species Bombyx mori [43]. It has been shown that silk fibroin is very promising for designing innovative drug delivery systems due to its excellent adhesion to tissues and living cells. Such properties make this material a promising substance for designing mechanisms of drug delivery that require high biocompatibility and effective interaction with biological tissues [44,45].

In 2020, Pan et al. [46] utilized PLGA in conjunction with the coaxial electrospray technique to create nanoparticles capable of simultaneously delivering dexamethasone and melatonin for glaucoma treatment. These nanoparticles were characterized by a size of 468 nm and a negative zeta potential of −21.7 mV. In vitro release studies demonstrated sustained release of the two drugs up to 15 days without any initial burst release. Cytotoxicity tests on R28 cells have shown these formulations to be non-toxic, mirroring what was seen in the control group. Melatonin-loaded nanoparticles reduced the intra-ocular pressure in in vivo tests on rabbits up to 120 h, wherein the reduction during the first 50 h was more pronounced with more sustained release rates from the nanoparticles that enhance the penetration in comparison to the free drug [37]. In another recent study by Dahiya et al. in 2023, chitosan-coated PLGA nanoparticles for the topical delivery of rutin and forskolin were prepared by emulsification and evaporation techniques [46]. These nanoparticles were of average size 136.64 ± 5.74 nm with a positive zeta potential of +34 mV. In vitro release tests indicated a continuous release of 70.87 ± 3.45% of the loaded forskolin and 65.14 ± 3.04% of the loaded rutin over 24 h, also improving permeation and tissue adhesion [46].

Elhabal et al. [47] developed a sustained bisoprolol loaded PLGA nanoparticles using a solvent displacement method. The optimal formulation demonstrated 75% drug encapsulation, a ZP of −18.7 ± 0.41 mV, a particle size of 105 ± 0.35 nm and the PI of 0.411 ± 0.71. In vitro evaluation showed an extended drug release for more than 12 h. In vivo studies on albino male rabbits, shows that the nanoparticles exhibit prolonged IOP reduction compared to drug solution with their maximum effect (70%) at 14 h which stays above 50% until 20 h [47].

Kandav et al. [48] created citicoline-loaded chitosan–gellan gum nanoparticles (CT-CS-GGNPs) with an ionotropic gelation process. The formulation was optimized by crosslinking chitosan (CS) and gellan gum (GG), yielding nanoparticles with physicochemical properties ranging from 241.3–990.2 nm for particle size, 12–83.33% for encapsulation efficiency (%EE), 0.302–0.978 for polydispersity index (PDI), and 1.23–35.2 mV for zeta potential. The improved formulation showed sustained in vitro drug release, releasing around 70.6 ± 1.9% of the medication over 12 h. Furthermore, ex vivo HET-CAM and corneal permeation tests verified the formulation’s non-irritant and biocompatibility. Notably, the nanoparticles dramatically increased corneal absorption, resulting in approximately 2.2-fold greater drug permeation than the usual drug solution [48].

Maria et al. [49] designed nimodipine-loaded chitosan nanoparticles (NMD-CS NPs) using the spontaneous emulsification solvent diffusion process with three different chitosan types: carboxymethyl chitosan (CMCS), low molecular weight chitosan (LCS), and medium molecular weight chitosan (MCS). The drug loading efficiency differed significantly among formulations, with values of 37.22 ± 1.28%, 7.52 ± 0.19%, and 14.55 ± 0.19% for NMD-CMCS, NMD-LCS, and NMD-MCS nanoparticles, respectively. In vitro investigations using CMCS, LCS, and MCS nanoparticle eye drops showed sustained drug release over 72 h, with cumulative release percentages of 45.3 ± 2.9%, 54.1 ± 1.7%, and 65.5 ± 4.8%, respectively. Particle size is one of the main causes of observed variances in release behavior. While the larger particle size of CMCS nanoparticles contributed to slower release, smaller particles, like those in MCS and LCS formulations, give a bigger surface area, resulting in faster drug release [49].

Hosmani et al. [50] designed poly(2-ethyl-2-oxazoline)-coated solid lipid nanoparticles (PSLNPs) with aa mean particle size of 107 ± 17 nm, a zeta potential of −49.6 mV, and a high entrapment efficiency of 96.2 ± 0.85%, indicating stable and efficient drug loading. In vitro investigations showed a biphasic release profile, with an initial burst followed by steady drug release over 12 h, resulting in a cumulative release of 81.65 ± 1.94%. This behavior points to an initial release of surface-associated drug, followed by regulated diffusion from the lipid matrix. Ex vivo permeation experiments revealed considerably increased transcorneal and trans-scleral drug transport relative to the marketed formulation. Furthermore, HET-CAM research validated the formulation’s non-irritant and biocompatible properties. In vivo studies revealed a more immediate, and prolonged IOP-lowering effect than the traditional formulation. The marketed product reduced IOP to 20.9 ± 0.6 mmHg at 4 h post-administration and gradually lost efficacy, with values approaching baseline (27.7 ± 0.5 mmHg) by 24 h. However, the PSLNP formulation had a faster onset of action, lowering IOP to 19.9 ± 0.5 mmHg within 1 h and reaching a minimum of 17.2 ± 0.5 mmHg at 4 h. During the 24 h investigation, clinically relevant IOP levels (<22 mmHg) were maintained [50].

The studies reviewed show that how well drugs are delivered to the eye depends on several factors, including particle size, surface charge, mucoadhesion, and formulation design; no single factor determines performance. Nanoscale carriers typically improve precorneal residence and drug availability by increasing surface contact and reducing clearing by tears, as seen in polymeric and lipid-based nanoparticle systems [27,42]. However, smaller particle sizes do not always lead to better results; quick drug release or low retention can limit potential benefits from better penetration.

Surface charge, especially the positive zeta potential in chitosan-based systems, is important for enhancing mucoadhesion through electrostatic interactions with the negatively charged mucin layer [27,46]. This increases residence time and local drug concentration, but too much mucoadhesion can slow down drug transport across the epithelium. This issue has been approached with hybrid systems like nanoparticle-in-gel formulations and gelatin–HPMC platforms, which find a balance between adhesion and controlled drug release [27,30].

In these systems, sustained drug release mainly results from diffusion through polymeric matrices or reservoir-type nanocapsules. These are often combined with increased viscosity or in situ gelation to extend ocular residence [27,30]. At the same time, better corneal penetration mainly stems from longer residence time, improved interaction with the epithelium, and enhanced drug solubilization rather than direct transport of nanoparticles [31,42].

The results from these studies highlight the capability of designed nanoparticles to achieve sustained drug release over periods exceeding 12 h. Incorporating these nanoparticles into in situ gelling systems or gels has further optimized the drug release profiles. At the same time, their inclusion has enhanced tissue adhesion and, consequently, the persistence of the drug effects in the eye. The use of coating materials such as chitosan and collagen on nanoparticles has not only improved their release profiles but also their tissue adhesion properties. Toxicity and ocular sensitivity evaluations show the high safety and biocompatibility of these nanoparticle-based formulations.

According to the outcomes of these studies, nanoparticles present a promising strategy for the sustained delivery of medications in treating ocular diseases, including glaucoma. Nanosystems offer advantages over traditional eye medications, including better drug absorption, improved bioavailability, less degradation of unstable drugs, longer ocular retention, greater corneal contact, and potential for targeted delivery. Smaller particles, such as nanoparticles, exhibit excellent tolerance and possess mucoadhesive properties, which contribute to the prolonged contact time of a drug with ocular tissue and enhance its bioavailability [51].

#### 3.2.2. Micelles

Micelles, due to their ability to provide continuous and sustained drug release, improve the solubility and bioavailability of drugs that are insoluble in water, have been recognized as an effective nanodrug delivery system for targeting various body tissues, including the eye [52]. It has also been utilized in various studies to attempt to achieve sustained drug release for more than 12 h. Of interest among these, ocular micelles have been developed to encapsulate various active pharmaceutical ingredients intended for delivery to the eye.

Among these are micelles containing latanoprost [53], dorzolamide [54]. Like nanoparticles, micelles exhibit low stability. Additionally, micelles have low drug-loading capacity. Their characteristics are also dependent on the critical micelle concentration [55].

In 2020, poly (lactide)-block-poly (methacrylic acid-co-3-acrylamidophenylboronic acid) block copolymer micelles were prepared by free radical polymerization and used as a carrier to formulate latanoprost for enhanced drug delivery to the eye. The in vitro release profile of latanoprost from these polymeric micelles was studied in simulated tear fluid using dialysis tubing. About 80% of the drug release from these micelles was observed on approximately day 12 in a pseudo-zero-order kinetic pattern. Results show an EE% of 23.7 ± 1.2%. The IOP-raising effect of the micellar latanoprost formulation was studied in genetically modified mice and an appreciable rise in IOP was noted in mutants compared to the control group, thus assuring that the raised IOP was due to conditions other than the micellar formulation. Importantly, mice treated with the micellar formulation of latanoprost exhibited a consistent reduction in IOP for 15 days compared to mice treated with latanoprost alone, demonstrating the enhanced efficacy of the micellar system in delivering latanoprost and reducing IOP effectively, which can provide a more sustained release of latanoprost, potentially offering a more effective treatment option for conditions such as glaucoma [53].

In an investigation published in 2023, polymeric micelles (PMs) were prepared by the dialysis technique of biocompatible graft copolymers comprising PCL and PCL-g-P(NVCL-co-NVP). This paper discussed the encapsulation of two different pharmaceuticals into these polymeric micelles. The micelle size increased after the encapsulation of dorzolamide, compared with the reduction in size observed post-encapsulation of indomethacin. Dynamic light-scattering tests for stability proved that both the blank and the drug-loaded micelles remained stable for 30 days at 4 °C. Uniformity and cytocompatibility of these micelles were confirmed during in vitro studies, hence providing enough grounds for being suitable for in vivo studies. The micellar system demonstrated its potential to facilitate the sustained release of encapsulated pharmaceuticals over a 24 h duration in vitro. Furthermore, in vivo investigations conducted on laboratory animals underscored the effectiveness of the micelles in decreasing IOP within 15 days [54].

Similar to nanoparticles, small size and amphiphilic structure enhance the solubilization of hydrophobic drugs and improve interaction with the corneal surface. Strategies for functionalization, such as the incorporation of phenylboronic acid, further enhance mucoadhesion and retention. However, micelles frequently exhibit limited structural stability in the tear film, leading to early drug release and a reduced duration of effect.

Micelles integrated into in situ gelling systems or hydrogels represent one of the significant advances in modern drug delivery technology, especially for extending the duration of release and enhancing ex vivo drug penetration. This technique proved substantial benefits in ocular drug delivery, where the control of drug release and prolongation is critical for both the therapeutic effect and patient compliance with the therapy. An important outcome of all these micelle-based delivery systems is the significant improvement in the safety profile of drugs known to be harmful, such as cyclosporine. By avoiding the initial burst release that is typical of more traditional emulsion-based delivery systems, micelles ensure that cells and tissues are not subjected to abrupt, high concentrations of the drug. This controlled release mechanism minimizes toxicity and enhances drug tolerability, making micelles an attractive option for ocular applications. Despite all the benefits, there are some drawbacks that make micelles a difficult choice for scaling up, including irritation due to the amphiphilic polymers or surfactants used in their formulation.

#### 3.2.3. Liposomes

Liposomes represent a complex delivery system for ocular pharmacological agents in their ability to encapsulate lipophilic and hydrophilic drugs due to their unique colloidal bilayer membrane structure [24]. Their useful properties include protecting drugs from degradation, high biocompatibility, and the possibility of targeted delivery to specific parts of ocular tissues. More importantly, positively charged liposomes penetrate more effectively and remain longer in ocular tissues [56]. Despite these positive features, however, liposomes possess certain disadvantages. The major drawbacks related to liposomes concern their poor stability, the relatively low drug entrapment efficiency, and the fast release of hydrophilic drugs [57]. The hydrolysis or oxidation of unsaturated lipids within standard liposomes leads to their chemical instability. Physical instability arises from the aggregation of liposome particles and drug leakage. Moreover, liposomes are susceptible to phagocytosis by phagocytes [58]. Despite these drawbacks, liposomes have been successfully utilized to deliver a variety of ocular drugs, including azithromycin, timolol, latanoprost, brimonidine, travoprost, brinzolamide, and bevacizumab [24,59,60,61,62].

In 2015, Fathalla et al. [62] prepared latanoprost-loaded liposomes using the method of reverse phase vaporization with the aim of developing ocular delivery. Such liposomes were formulated in gels containing carbopol 934, Pluronic 127 and HPMC polymers for ocular delivery. The size of the produced liposomes was measured between 0.99 and 1.30 μm. Fitting the in vitro drug release profiles, it was found that the liposome suspension, carbopol gel containing liposomes, and HPMC gel with liposomes gave similar release patterns, each releasing about 40% of the drug within 24 h. In contrast, the introduction of liposomes into the Pluronic gel reduced the rate of drug release to about 30% in the same period, which could be due to the high viscosity of the gel and hydrophobic interactions between Pluronic and latanoprost that sustained the release of the drug. Drug release from all the prepared systems was extended for approximately 50 h. An irritation study conducted in rabbit eyes using Pluronic gel containing latanoprost liposomes showed no signs of either toxicity or irritation, thus demonstrating the great safety profile of the formulation. When comparing the IOP-lowering efficacy of the Pluronic gel containing latanoprost liposomes with traditional latanoprost eye drops in rabbits, both treatments rapidly reduced eye pressure, achieving maximum reduction within 4 h. However, the formulated gel provided a more substantial reduction in IOP than the eye drops. Moreover, while ocular pressure reverted to its baseline level 24 h after administering the eye drops, the pressure remained normal up to 72 h following the application of the gel formulation [62].

In 2019, Hathout et al. [61] prepared gel core liposomes containing timolol maleate, aiming to provide a sustained drug delivery system. The vesicles were prepared by a thin-layer hydration method, one of the most widespread methods for the preparation of liposomal formulations. By using this method, it was possible to prepare liposomes capable of providing, in a sustained way, the release of the encapsulated drug up to 24 h, as shown by in vitro experiments. This technique not only improved the encapsulation of timolol maleate to as high as 50% but also maintained a comparatively small particle size of 38.81 μm, which is desirable for ocular drug delivery. The in vivo evaluation of these gelatinized core liposomes was highly effective in reducing IOP for 24 h. Moreover, histological analyses validated the biocompatibility of these liposomes, revealing no negative tissue responses [61].

Qiu et al., in 2023, [59] designed a new oligoethylenimine-modified dendritic liposome for the simultaneous delivery of two anti-glaucoma drugs: latanoprost and timolol. 1,2-Distearoyl-sn-glycero-3-phosphoethanolamine-Poly (ethylene glycol) (DSPE-PEG) and oligoethylenimine (OEI) were the main polymers in the preparation of the liposomes. The new lipoformulation had a uniform nanoscale size, a positive surface charge, and an excellent dual drug encapsulation capacity that may be considered one of the significant improvements in ocular delivery systems. Increased cellular uptake, mediated by oligoethylenimine modification, and the system’s ability to disrupt tight junctions dramatically increase intercellular and paracellular transport through the corneal structure. The in vitro study presented here shows sustained-release profiles of the two drugs entrapped in several types of liposomal formulations. Accordingly, it was possible to evidence that timolol released relatively fast from negative liposome, positive liposome, and timolol and latanoprost co-loaded PL with total release times of 12, 32, and 28 h, respectively. The obtained findings suggest that charge features and the co-loading strategy of liposomes play an important role in modulating timolol release kinetics. The release of latanoprost from liposomes was much slower when compared with timolol, reflecting differences in the nature of the drugs and in their interactions with the liposomal components. In the case of negatively and positively charged liposomes, about 75% of latanoprost was released in 144 h, indicating a prolonged release over a long period. In vivo studies with Norwegian brown rats showed the immense effectiveness of this liposomal drug delivery system. A single dose of liposome formulation with combined latanoprost and timolol induced a steady and extended reduction in IOP up to five days. Significantly, such therapeutic efficacy was successfully delivered without the induction of inflammation or irritation or causing any ocular tissue damage [59].

In contrast to micelles, liposomal systems provide greater structural flexibility, allowing for the encapsulation of both hydrophilic and lipophilic drugs, and enabling surface modification to enhance retention and permeability. Cationic and dendritic modifications have been found to improve corneal interaction, while hybrid systems such as liposomal gels and gelatinized-core liposomes introduce additional diffusion barriers that support sustained release. Nevertheless, liposomes remain susceptible to physical instability and drug release issues, which may compromise long-term performance.

**Table 2:** Overview of Ocular Anti-Glaucoma Delivery Using Nanoparticles, Micelles, and Liposomes.

Drug	Drug Delivery System	Polymer	Method	Zeta Potential (mV)	Particle Size	In Vitro Release Time	In Vivo Animal and Results	Important Results	References
Brimonidine	Nanoparticle/in situ gel	Vit E/TPGS/PCL Poloxamer 407	Nanoprecipitation/cold method	3.23	243.4 nm	24 h	IOP reduction: Rabbit eye for 24 h transcorneal permeation: gout eye	IOP reduction activity of nanoparticle/in situ gel: 2 > solution	[27]
Pilocarpine	Nanoparticle	PCL	Emulsion–solvent evaporation	-	-	40 d	IOP reduction in the rabbit eye continued till 42 d after injection	Drug loading in nanocapsules is more than nanospheres.	[28]
Timolol	Nanoparticle	Gelatin	Double desolvation	12.5	205 nm	25 h	IOP reduction in rabbit eyes continued for 25 h	-	[29]
Timolol	Nanoparticle/gel	Gelatin/HPMC	Ethanol–water solvent displacement	−0.684	193 nm	4 d	IOP reduction in rabbit eyes lasted for 12 h	Incorporation of nanoparticles in gel improved the release time of the timolol from the formulation.	[30]
Pilocarpine	Nanoparticle	Ceria/CS/ZM241385	Sol–Gel method	12.5–36.3	-	7 d	IOP reduction in rabbit eyes lasted for 3 d	Coating of nanoparticle with chitosan and ZM241385 increased drug loading.	[31]
Pilocarpine	Nanoparticle	ZnO/PVP/collagen	Solvent casting method	23.2	20 nm	14 d	-	Collagen improved the mechanical and mucoadhesive properties of nanoparticles.	[32]
Dexamethasone/melatonin	Nanoparticle	PLGA	Co-axial electrospray	−21.7	468 nm	15 d	IOP reduction in rabbit eyes lasted for 120 h	Burst released has not been reported.	[37]
Betaxolol hydrochloride	Nanoparticle	Chitosan	Spontaneous emulsification	25.2–26.4	168–260 nm	12 h	IOP reduction in rabbit eyes lasted for 5 h	Stability tests of the nanoparticles indicated that no notable change in particle size or drug content after 3 months of storage at 25 ± 2 °C and 60 ± 5% relative humidity was observed.	[38]
Brinzolamide	Lipid–polymer nanoparticles	PLGA	Oil in water (o/w) emulsification–solvent evaporation	7.53	151.23 nm	12 h	IOP reduction in rabbit eyes lasted for 24 h.	Relative cumulative corneal permeability of Brinzolamide from LPNs was much higher compared to the commercially available AZOPT^®^.	[42]
Rutin, Forskolin	Nanoparticle	PCL, PLGA	Emulsification, evaporation	+34	136.64 nm	24 h	-	Nanoparticles improved corneal permeation and mucoadhesiveness.	[46]
Latanoprost	Micelle	Poly (lactide)-, poly (methacrylic acid-co-3-acrylamidophenylboronic acid)	Free radical polymerization	-	-	12 d	Mice treated with the micellar formulation exhibited a consistent reduction in IOP for 15 days	-	[53]
Dorzolamide, Indomethacin	Micelle	PCL and PCL-g-P(NVCL-co-NVP)	Dialysis technique	-	39–47 nm	24 h	Decreasing IOP within 15 days	Dynamic light-scattering tests for stability proved that both the blank and the drug-loaded micelles remained stable for 30 days at 4 °C.	[54]
Latanoprost	Liposomes	Carbopol 934, Pluronic 127 and HPMC	Reverse phase vaporization	-	0.99 and 1.30 μm.	50 h	Decreasing IOP within 3 days	The formulations rapidly reduced eye pressure, achieving maximum reduction within 4 h	[62]
Timolol	Gel core liposomes	Cholesterol, gelatin	Thin-layer hydration	-	38.81 μm	24 h	IOP reduction in rabbit eyes lasted for 24 h	Histological analyses validated the biocompatibility of these liposomes, revealing no negative tissue responses	[61]
Timolol and Latanoprost	Oligoethylenimine-modified dendritic liposome	DSPE-PEG, OEI	-	24.3–24.7	100–120 nm	32 h for timolol and 144 h for latanoprost	IOP reduction in Norwegian brown rats lasted for 5 days	The components of the ocular tissue show no clear structural or pathological abnormalities.	[59]

#### 3.2.4. Nanofibers as Ocular Insert

Fibers, which can be defined as highly elongated structures with a comparatively low diameter, possess high flexibility [63], tend to be obtained from polymer solutions or their melts by mainly two ways: electrospinning and drawing. For a given polymer, the various processing conditions employed to produce the fiber are influenced by, among others, time and temperature-dependent molecular movements, phase transitions under tension, entanglement limits, and several types of intermolecular reactions. It has been discovered that as many as process variables, which include stress, strain, temperature, time and the size and distribution of molecules, affect the final properties and morphology of the fibers. Out of the overwhelming amount of nanofiber production methods published in the literature, five principal methods are put forward: drawing, electrospinning, phase separation, template synthesis, and self-assembly. In this regard, electrospinning is highlighted as the only method available for nanofiber production on an industrial scale [64]. Various polymer dispersions such as PLGA, PCL, PLA, chitosan, Eudragit, ethyl cellulose, collagen etc., could also be used in this process [65]. Nanofibers are particularly considered for ocular drug delivery due to their benefits. The high degree of interconnection within nanofibers allows for certain sustained drug release profiles in it. This slow-release ability would reduce the frequency of necessary dosing, which may improve compliance [66]. Nanofiber inserts have been studied for the delivery of various drugs, for instance, dexamethasone, a combination of gentamicin and methylprednisolone, azithromycin, and triamcinolone [67]. Studies related to nanofibers and ophthalmic inserts capable of sustained drug release for more than 12 h are summarized in Table 3. According to numerous studies, the use of ophthalmic nanofibers can lead to crystal vision, eye irritation, and watery discharge [68]. These issues and limitations can be alleviated by optimizing the size and surface charge of nanofibers, combining them with other drug delivery systems, and using suitable polymers (Table 4 and Table 5) [69].

For the management of glaucoma, nanofibers have been used for single and dual delivery of different drugs such as timolol, dorzolamide and brimonidine.

Cooley invented the electrospinning technique that was patented in 1900. Electrospinning is a very cost-effective technique for making very thin fibers from polymer solutions. This technique has since found numerous applications in the medical field, including drug delivery using nanofibers, fabrication of scaffolds for tissue engineering, development of membrane filters, biosensors, and emulsifying enzymes. The electrospinning process employs a potent electric field, typically ranging from 1 to 30 kV. A polymer solution or melt polymer is loaded into a syringe, and upon applying a high-voltage electric field, the polymer droplet suspended from the syringe tip is influenced by the field. This results in the distribution of induced charges across the droplet’s surface, causing it to elongate into a cone-like shape known as the Taylor cone. Once the electric force surpasses the surface tension of the droplet, one or more charged jets eject from the tip of the droplet towards a collecting plate. The fibers deposit on the metal collector plate when the solvent evaporates. Factors such as temperature, humidity and the viscosity of the polymer solution affect both the efficiency and quality of the electrospinning process to a great extent [65]. Advancement within the field has led to the invention of advanced electrospinning techniques that comprise coaxial electrospinning, emulsion electrospinning and the use of double or multi syringes. These endeavors are intended to improve drug stability against degradation and to optimize the drug release profiles. Specifically, these methods facilitate the fabrication of nanofibers with a core–shell structure, offering new possibilities in the sustained delivery of therapeutics [70].

In 2022, as reported by Mirzaeei et al. [71], sustainable ophthalmic nanofibers containing timolol for the treatment of glaucoma were developed using the electrospinning technique with PCL, Eudragit RL100 and cellulose acetate polymers. The diameter of the produced nanofibers was in the range of 104 to 176 nm. The formulations developed had good physicochemical properties and stability appropriate for delivery into the eye. In vitro drug release studies showed sustained drug release from PCL nanofiber for 3 days. These formulations, when administered, did not produce significant signs of irritation or any damage to the tissues. The IOP-reducing effect of this nanofiber in horses with IOP above 27mmhg demonstrated that nanofibers containing timolol lowered IOP within six days [71].

Shoushtari et al. [72] prepared brimonidine-loaded nanofibrous inserts in 2024 using various polymers such as poly (D, L-lactide), PCL, cellulose acetate, and Eudragit RL100^®^, loading brimonidine via the process of electrospinning. SEM images revealed that the nanofibers had uniform morphology with an average diameter of less than 300 nm. In the BMD-PCL-EUD formulation, the addition of EUD to PCL enhanced the swelling as compared to the PCL-only formulations. The degree of swelling for the BMD-PCL-EUD mixture was 187.7 ± 5.3% in 24 h. These nanofibers demonstrated high strength and sufficient flexibility for placement in the conjunctival sac. FTIR confirmed drug–polymer compatibility. In vitro release studies demonstrated a sustained drug release pattern over a period of 6 days. In vivo assessments revealed that the optimized formulation effectively maintained IOP within a normal range for a prolonged period of 6 days, and was found to be non-irritating to caprine eyes [72].

In the study conducted by Tarun Garg et al. [73], an ophthalmic nanofiber with incorporated timolol and dorzolamide was prepared using PVA and PCL polymers via the electrospinning technique. The in vitro drug release analysis indicated that these nanofibers could sustain drug release up to 24 h; the PCL nanofibers released the drug more slowly compared to the PVA nanofibers, probably due to the hydrophobic nature of PCL and a lower swelling capacity. By means of SEM, the diameter of nanofibers was around 200–400 nm and had a rather smooth surface. The swelling ratio varied within the range from 180 to 110%. Since PVA nanofibers are smaller in size and more hydrophilic compared to PCL, they swell more. The study of their efficacy in the reduction in IOP in rabbits revealed that PVA and PCL nanofibers attained their peak effect at 16 and 20 h, respectively, sustaining this pressure-reducing effect for as long as 72 h. In addition, ocular irritation was investigated by the Draize test. The results showed that the PCL nanofibers induced mild eye redness; this was probably because of their hydrophobic nature and ionic properties [73].

A nanofiber-based ocular insert containing timolol (TIM) and dorzolamide (DOR) was designed by Karami et al. with Eudragit RL100 (EUD) as the polymeric matrix. The developed nanofibers had a mean fiber diameters less than 465 nm. In vitro release experiments showed good drug retention and sustained release of both TIM and DOR for up to 80 h. Biocompatibility tests revealed good cell viability, and ocular irritation experiments indicated that the formulation was non-irritating in rabbit models [74].

In contrast to nanoparticle systems, nanofiber-based systems function as macroscopic drug carriers designed for extended release over prolonged periods. Electrospun nanofibers enable controlled drug release through diffusion and polymer degradation, maintaining therapeutic levels for days or weeks while minimizing rapid clearance. This makes them particularly suitable for extended-release applications exceeding conventional topical systems. However, their clinical use is influenced by factors such as patient comfort, foreign body sensation, and the need for proper placement or removal, which may limit widespread acceptance. Nanoparticle-based systems, such as liposomes and polymeric nanoparticles, are distinguished by their nanoscale size, which allows for improved penetration through ocular barriers and enhances drug absorption at the targeted site. This characteristic leads to greater bioavailability and elevated therapeutic effectiveness. However, this same capability to move across biological membranes may also cause unintended distribution away from the intended action site, which could raise the risk of off-target effects and systemic exposure [75,76]. In contrast, nanofiber-based systems display different behavior due to their structure, characterized by a high aspect ratio and elongated length in one dimension. Instead of penetrating ocular tissues, nanofibers usually stay localized at the administration site, which facilitates a longer residence time and decreases clearance by tear turnover.

Additionally, nanofibers exhibit distinct mechanical properties, such as flexibility and structural stability, enabling them to act as reliable ocular inserts that maintain their position within the eye. Moreover, the release of drugs from nanofiber matrices is usually controlled by diffusion and the degradation of polymers, leading to a more consistent and prolonged release profile that can last from several days to weeks [71,72,73].

**Table 3:** Summary of nanofiber and insert-based anti-glaucoma delivery.

Drug	Formulation	Method	Polymer	Swelling	Diameter	Thickness	Tensile Strength	In Vitro Release Time	In Vivo Animal and Results	Ref.
Timolol/Dorzolamide	Nanofiber	Electrospinning	PVA_PCL	110–180%	200–400 nm	-	-	24 h	Maintaining the IOP for up to 72 h in rabbit’s eyes.	[73]
Timolol	Nanofiber	Electrospinning	PCL, cellulose acetate, Eudragit RL100	150.3–188.2%	104–176 nm	0.087–0.408 mm	0.46–50.4 Mpa	3 d	No irritation and toxicity to the equine eyes while decreasing the IOP for 6 days.	[71]
Brimonidine	Nanofiber	Electrospinning	PDLLA, PCL, CA, Eudragit RL100	187.7 ± 5.3%	>300nm	<0.300 mm	-	6 d	Maintaining the IOP in a non-glaucomatous range for a duration of 6 days in caprine eye.	[72]

PDLLA = Poly(DL-lactide), CA = Cellulose acetate.

**Table 4:** Advantages and drawbacks of novel drug delivery systems.

Drug Delivery System	Advantages	Drawbacks	References
Nanoparticles	Polymeric nanoparticles are capable of targeting both segments of the eye. They provide sustained drug release over time. They improve drug permeation across ocular barriers. They decrease the rate of drug elimination, leading to better therapeutic outcomes. Their nanoscale size enhances patient adherence, especially for long-term ocular conditions.	In polymeric nanoparticles, polymer degradation may lead to harmful systemic effects. The use of organic solvents in the final formulation can also contribute to safety concerns.	[77]
Micelles	Micelles are capable of encapsulating both hydrophilic and lipophilic drugs. They are prepared through relatively simple techniques. They improve drug bioavailability. They are able to deliver drugs effectively to both the anterior and posterior segments of the eye.	A key drawback of micellar carriers is the potential ocular irritation caused by the amphiphilic polymers or surfactants used in their formulation. Another limitation is the difficulty in scaling up their production for larger manufacturing processes.	[52]
Liposomes	Liposomes possess biodegradable properties. They can encapsulate both hydrophilic and lipophilic medications They can be prepared using simple fabrication methods. They enhance the bioavailability of drugs.	Liposomes can undergo chemical instability from hydrolysis or oxidation of their unsaturated lipid components. Physical instability may occur due to leakage of the encapsulated drug. Aggregation of liposomes into larger particles can reduce ocular absorption Larger aggregates are also more prone to phagocytosis by immune cells. Lack of scalability potential owing to its low stability and high production cost	[58,78]
Nanofibers	Nanofibers possess a large surface area. They feature a highly porous structure. They can be fabricated using a variety of polymers. They offer a high drug-loading capacity. Nanofibers, in particular, have more flexible diameter control compared to other carriers, enhancing drug permeation through ocular tissues. They can be integrated with other drug delivery systems for improved therapeutic outcomes. Due to its water-free base, it does not require preservatives	Possible side effects include blurred vision, eye irritation, and watery discharge. Side effects can be reduced by optimizing nanofiber characteristics such as size, surface charge, polymer type and composition.	[69]

**Table 5:** Comparison of different novel ocular drug delivery systems.

Drug Delivery System	Drug Loading Capacity	Surface Area & Porosity	Sustained Release	Diameter/Size Adjustability	Stability	Polymer/Material Flexibility
Nanofibers	Very high (large surface area and porous structure allow more drug incorporation)	Large surface area, highly porous → improves release and permeation	Excellent, tunable via fiber diameter and polymer selection incorporation)	Easily adjustable fiber diameters (better tissue permeation)	High mechanical and structural stability	Wide range of natural/synthetic polymers
Nanoparticles	Moderate (limited by particle core size)	Moderate surface area, not porous	Good, but sometimes limited by burst release	Restricted to nanometer-scale	May aggregate or crystallize	Dependent on polymer/solvent used
Micelles	Low–moderate (mainly hydrophobic drugs in micelle core)	Small hydrophobic core; limited surface	Less sustained, faster release	Restricted to nanometer-scale	Prone to leakage	Requires amphiphilic surfactants
Liposomes	Moderate (lipophilic drugs in bilayer; hydrophilic drugs in aqueous core) drug incorporation)	Moderate, but prone to aggregation	Good, but stability issues (leakage, fusion)	Restricted to nanometer-scale	Prone to chemical/physical instability (hydrolysis, oxidation, leakage)	Limited to lipid-based components

## 4. Translation to Clinic

Despite promising results in early studies, using extended-release ocular drug delivery systems in clinical settings requires addressing several important milestones. This includes detailed in vitro and in vivo assessments, followed by standardized biocompatibility tests according to ISO 10993-6 guidelines [79] for ocular applications. These tests look at local tissue responses, such as inflammation, irritation, and structural changes after implantation. Before clinical trials, these systems must meet regulatory requirements for Investigational New Drug (IND) submissions. This covers information on pharmacokinetics, toxicity, and manufacturing consistency. Additionally, considerations about scaling up production and costs are crucial for evaluating the feasibility of introducing these products to the market. Although nanocarrier-based systems offer significant advantages in drug delivery effectiveness, challenges related to regulatory approval, manufacturing complexities, and economic viability must be carefully examined to facilitate their transition from lab research to clinical use.

Significant research has been conducted on nanocarriers such as nanoparticles and liposomes for the treatment of glaucoma. They are undergoing clinical trials and have demonstrated favorable patient results. The POLAT-001 study was a Phase II, randomized, open-label, multicenter clinical trial designed to evaluate the safety and efficacy of a subconjunctival liposomal latanoprost formulation (POLAT-001) compared with conventional latanoprost ophthalmic solution (0.005%) in patients with primary open-angle glaucoma [80]. A total of approximately 80 patients were randomized to receive either a single subconjunctival injection of POLAT-001 or daily topical latanoprost eye drops, with follow-up assessments extending to three months.

Patients treated with POLAT-001 demonstrated a mean IOP reduction of −2.3 mmHg (SD 4.6), whereas those treated with topical latanoprost achieved a more pronounced reduction of −6.4 mmHg (SD 2.9). These findings indicated that, under the study conditions, POLAT-001 was less effective than standard topical therapy in lowering IOP.

In terms of safety, POLAT-001 was generally well tolerated, with no systemic adverse effects reported. The most common ocular adverse effects in the injection group included conjunctival hemorrhage (26.4%), foreign body sensation (17.0%), and conjunctival hyperemia (13.2%).

Although earlier pilot studies of liposomal latanoprost had suggested substantial and sustained IOP reductions of up to 47% over three months [81], the larger Phase II trial did not replicate these outcomes. Differences between experimental models and real-world settings are the primary cause of discrepancies between preclinical and clinical results in ocular drug delivery systems, like POLAT-001. Because of their simpler ocular physiology and fewer clearance systems, animal studies frequently overstate efficacy, whereas human eyes provide more dynamic environments. Ophthalmic drug delivery research frequently uses animal models; however, they have a number of drawbacks that may make it difficult to apply findings in clinical settings. The human eye and often used models (such as rabbits) differ significantly in terms of anatomy and physiology, including variances in tear turnover, blinking rate, corneal thickness, and ocular surface composition [82,83]. Preclinical studies frequently overestimate drug retention and bioavailability as a result of these discrepancies. Furthermore, the complexity and variety of human ocular disorders, such as glaucoma development and patient-specific variables, are generally not fully replicated in animal models. Pharmacokinetic behavior may also vary due to differences in drug distribution and clearance mechanisms. Furthermore, physiological conditions may alter formulation stability and drug release behavior, resulting in decreased drug availability. Translation is further restricted by differences in pharmacokinetics, dose scaling, and drug distribution. Lastly, sustained release alone is insufficient without significant therapeutic performance because clinical trials require equivalent or greater efficacy to existing treatments.

The intracameral bimatoprost implant (Durysta^®^) was studied in large Phase III trials (ARTEMIS 1 and 2) with over 1100 patients. It decreased IOP by about 6–8 mmHg, similar to the effects of topical timolol twice daily [84,85]. The implant released the drug steadily for around 4 to 6 months after a single dose, and the study monitored patients for up to 20 months. Similarly, travoprost intracameral implants, such as iDose TR, were tested in Phase II and III trials involving more than 1100 patients [86]. They sustained IOP reductions of 6 to 8 mmHg over 6 to 12 months, matching the effects of twice-daily topical therapy. However, both implant systems have procedure-related issues and safety concerns. With Durysta^®^, there is a risk of corneal endothelial cell loss. For iDose TR, potential complications may require surgical placement and replacement of the implant.

On the other hand, less invasive systems like the travoprost punctal plug (OTX-TP) were studied in Phase II and III trials involving about 500 to 700 patients [87,88]. They provided modest, variable IOP reductions of approximately 3 to 5 mmHg over study periods of up to 3 months. Although these systems offered the advantage of less frequent dosing compared to topical therapies, which are given once or twice daily, their performance in clinical settings was inconsistent. This variability mainly arose from differences in plug retention and drug release. The side effects reported with OTX-TP were generally mild, including epiphora and a foreign-body sensation. However, problems such as plug displacement significantly reduced its effectiveness. Despite these advancements, several common challenges still limit the successful use of extended-release ocular drug delivery systems in everyday practice. These challenges include strict regulatory rules for complex combination products, difficulties in achieving consistent large-scale manufacturing, and technical issues with sterilizing new formulations.

Additionally, economic factors like production and treatment costs can affect access. Patient acceptance, especially for invasive methods, remains a major concern. Competition from existing therapies also makes adoption harder. Overall, these issues highlight the ongoing struggle to balance sustained drug release with effectiveness, safety, and practicality. They also emphasize the need to improve these delivery systems continuously.

In the case of advanced ocular drug delivery systems, key regulatory concerns typically include sterility, biocompatibility, classification of device and drug, manufacturing controls, stability, and evidence from clinical trials demonstrating that the system provides advantages over traditional formulations. Since many ocular systems function as implants, inserts, depots, prefilled applicators, or other combined systems, regulators may categorize them as drug–device combination products, which implies that both the drug and device components must adhere to their respective GMP/quality requirements [89].

A primary consideration is the quality of design and manufacturability. It is expected that sponsors will define the intended performance of the product, identify critical quality attributes, and implement a risk-based approach to manage variability in formulation and processes. In the case of ocular extended-release systems, this typically involves managing particle size or geometry, ensuring dose uniformity, controlling release kinetics, ensuring compatibility with container closure or devices, and maintaining consistent sterilization and assembly processes [90].

A second significant concern is sterility and stability, which are crucial for ocular products since they come into contact with sensitive tissues and, in some instances, are used in intraocular implants or injections. Regulatory authorities require a stability package that validates the proposed shelf life and confirms that product performance remains consistent without influencing clinical outcomes. For advanced ocular systems, this can involve the stability of the active ingredient, excipients, release characteristics, and the performance related to the device [91]. Throughout nanocarrier systems, the characteristics of formulations introduce additional regulatory and practical obstacles. Numerous nanoparticles, liposomal, and micellar formulations exist in the form of aqueous dispersions, which heightens the risk of microbial contamination and complicates the sterilization process, as traditional methods like heat sterilization might destabilize the carrier system or change the drug release profile. It also makes them susceptible to physiochemical instability. Consequently, these formulations frequently necessitate aseptic processing or the incorporation of preservatives to ensure sterility during storage and application, leading to concerns about ocular irritation and long-term safety [75,76]. In comparison, solid or semi-solid formulations like nanofiber-based inserts provide benefits in this area, as they can generally be sterilized with terminal techniques such as gamma irradiation or ethylene oxide without greatly affecting their structural integrity or drug release capabilities, and they are usually free of preservatives, which lessens the likelihood of toxicity linked to preservative use [71,73]. This structural stability also enables longer shelf life.

A third fundamental area is biocompatibility. ISO 10993-6 addresses the local effects following implantation, while ISO 10993-1 [92] identifies it as a component of the biological evaluation framework for devices. When it comes to ocular inserts, implants, or depot systems, regulators will be concerned about local inflammation, tissue injury, healing response, and material compatibility within the intended ocular location. ISO 10993-6 is particularly important when the material remains within the tissue or is implanted for prolonged release [79].

Another important factor to consider is the regulatory pathway itself. While a topical eye drop reformulation may generally adhere to a drug framework, devices such as implants, inserters, punctal plugs, or preloaded applicators can raise questions about combination products and introduce more complex chemistry, manufacturing, and controls (CMC) and human factors requirements. The FDA’s review documents for the travoprost intraocular implant categorize it as a combination product that includes a sterile implant preloaded in a sterile inserter, exemplifying how advanced ocular systems are assessed in practice [93].

Regulators prioritize clinical relevance in addition to extended-release capabilities. Typically, an advanced system must demonstrate not only prolonged drug release but also that it provides significant advantages, such as enhanced adherence, decreased treatment inconvenience, fewer administration, or clinically significant efficacy while maintaining an acceptable safety compromise.

## 5. Conclusions and Future Directions

Nanomedicine provides numerous advantages over conventional ophthalmic medications for effective ocular drug delivery, as it can enhance the therapeutic index by overcoming ocular barriers, improving drug release profiles, and reducing potential drug toxicity [94]. Their performance is heavily influenced by physicochemical factors including particle size and surface charge, which control ocular retention and drug diffusion. However, nanoparticles often exhibit poor stability and reproducibility, along with low drug entrapment efficiencies [95]. Micellar systems, on the other hand, primarily improve drug solubility and permeability, especially for hydrophobic medicines, and can demonstrate moderate sustained release via polymeric self-assembly and mucoadhesive interactions [53,54]. However, their lesser structural stability compared to nanoparticles may limit their ability to achieve long-term release. Liposomes provide a biocompatible lipid-based framework for encapsulating both hydrophilic and lipophilic drugs. Surface-modified liposomes have been shown to improve corneal retention and penetration while also providing prolonged drug release characteristics [59,61,62]. The primary disadvantages associated with liposomes also include their inadequate stability, relatively low drug entrapment effectiveness, and rapid release of hydrophilic drugs. The hydrolysis or oxidation of unsaturated lipids in typical liposomes results in chemical instability. Physical instability results from the aggregation of liposome particles and the leakage of the drug. Furthermore, liposomes are vulnerable to phagocytosis by phagocytic cells. Nanofiber-based systems, particularly electrospun inserts, provide the longest drug release profiles, often lasting several days to weeks, due to their high drug-loading capacity and matrix-controlled diffusion mechanisms [71,73,74]. These methods also greatly improve ocular residence time and pharmacokinetic metrics including AUC and mean residence time.

Nanofibers are advanced novel sustained drug delivery systems capable of administering multiple drugs for a prolonged duration. The dry nature of nanofibers contributes to their high physicochemical and microbial stability, eliminating the need for preservatives. Safety assessments, including biocompatibility studies conducted on rabbit eyes and various cell lines, further establish the safety of ocular nanofibers [69]. Thus, nanofibers can be a more stable and reliable novel drug delivery system, compared to other nano and microsystems. Compared to other drug delivery technologies, the electrospinning technology has significant versatility in the selection of materials and Active Pharmaceutical Ingredients (APIs) for release [96]. A notable advantage of this system is its anhydrous nature, which eliminates the need for preservatives. Additionally, advances in nanofiber manufacturing enable scalable production and potential industrial expansion in the future. Despite these advantages, the current level of clinical evidence is low. Furthermore, various practical issues must be addressed, including patient comfort, potential foreign body feeling, insertion and removal methods, retention stability, and long-term safety. These characteristics may have an impact on patient acceptance and real-world application. As a result, while nanofibers have significant potential, more clinical research is needed to thoroughly assess their efficacy, safety, and feasibility in comparison to present and upcoming ocular drug delivery systems. Overall, nanoparticles and liposomes offer a balance between efficacy and practicality, whereas nanofiber systems have a longer release duration, and micelles are largely solubility-enhancing carriers with moderate sustained-release capabilities. The choice of an acceptable system is thus determined by the required balance of release duration, stability, patient acceptability, and translational feasibility.

In conclusion, the future of eye condition treatments such as glaucoma looks promising with the advent of ocular nanocarriers such as nanofibers, nanoparticles and other innovative methods. These systems offer solutions to the challenges posed by traditional ocular formulations, providing more effective and efficient ways to administer treatments. Despite their potential, the commercial availability of these advanced drug delivery products remains limited for some of them. It is anticipated that ongoing research and development efforts will lead to the enhancement of these technologies, enabling the production of drug delivery systems that maintain their optical and physical properties over extended periods, thereby enhancing patient care and treatment outcomes. In this regard, the development approach for drug delivery methods should be such that they are capable of industrialization. For this purpose, systems that have the following characteristics are preferred: composed of biocompatible excipients, simple manufacturing processes, high drug loading with minimal wastage, simple and scalable production steps, compatibility with conventional sterilization methods, and easy, non-invasive administration techniques.

Translation of ocular drug delivery technologies from research to clinic presents numerous challenges: The eye possesses several protective barriers, including the corneal epithelium and blood–retinal barrier, which restrict medication penetration and bioavailability. Formulating effective medication delivery systems for targeted ocular tissues while ensuring stability and efficacy is intricate. Facilitating patient compliance with treatment protocols, particularly for chronic disorders such as glaucoma, can be challenging. Non-invasive delivery methods are favored; nonetheless, they frequently encounter constraints in efficacy. Navigating the regulatory framework for the approval of novel ocular pharmaceuticals and delivery mechanisms can be time-consuming and expensive. Scaling up production from laboratory to industrial scale while ensuring quality and consistency presents a considerable barrier. The substantial expenses associated with research, development, and clinical trials can slow the introduction of novel ocular drug delivery systems to the market. Furthermore, the utilization of pharmacokinetic studies can significantly reduce both the time and cost associated with formulation development and is recommended for future studies. The development of ocular dosage forms can be improved by pharmacokinetic research. This is expected to reduce the number of studies by providing guidance for the design of dosage forms and clinical trials. A precise pharmacokinetics model could proficiently resolve bioavailability challenges in ocular drug delivery development and may result in significant enhancements. Translation of advanced ocular drug delivery systems from research to clinic faces limitations that necessitate inventive strategies and ongoing research to overcome, ensuring that novel therapies can efficiently reach patients in need.

While the majority of current ocular drug delivery devices are intended to target the anterior segment, particularly for IOP reduction, glaucoma is primarily a neurodegenerative disease of the optic nerve and retinal ganglion cells in the posterior segment. Drug transport to the posterior portion remains much more difficult due to barriers such as the blood–retinal barrier and restricted permeability across ocular tissues. Although several nanocarrier systems, such as ceria nanoparticles and surface-modified liposomes, have shown promise for trans-scleral or deeper tissue penetration, their ability to achieve therapeutically appropriate concentrations in the posterior segment is limited. This demonstrates a fundamental gap in existing delivery options. Future research should concentrate on dual-targeting methods that address both anterior pressure regulation and posterior neuroprotection.

## Figures and Tables

**Table 1:** Topical drugs commonly utilized in the treatment of Open-Angle Glaucoma [10].

Generic Examples		Usual Dosage
1. Agents that reduce aqueous inflow		
β-Blockers	Betaxolol, Timolol, Carteolol, Levobunolol	1 drop BID or 1 drop daily or BID (for Levobunolol)
α2-Selective Adrenergic Agonists	Brimonidine, Apraclonidine	1 drop BID to TID for Brimonidine and 1 drop preoperatively and postoperatively or 1 drop BID to TID for Apraclonidine
Topical Carbonic Anhydrase Inhibitors	Brinzolamide, Dorzolamide	1 drop TID
2. Agents that enhance aqueous outflow		
Prostaglandin Analogs	Latanoprost, Travoprost, Bimatoprost, Tafluprost	1 drop once a day at bedtime
Cholinergic agonists (Miotics)	Pilocarpine, Carbachol	1–2 drops TID or QID
Rho kinase inhibitors	Netarsudil	1 drop once a day at bedtime
3. Combination Products		
Brimonidine tartrate 0.2%/timolol 0.5%	Sympathomimetic/sympatholytic	1 drop BID
Dorzolamide 2%/timolol 0.5%	Decreased aqueous humor production/sympatholytic	1 drop BID
Brinzolamide 1%/brimonidine 0.2%	Decreased aqueous humor production/sympathomimetic	1 drop TID

BID, twice daily; QID, 4 times a day; TID, 3 times a day.

## Data Availability

No new data were created or analyzed in this study.
